# Emotional Pain Mediates the Link Between Preoccupied Attachment and Non-suicidal Self-Injury in High Suicide Risk Psychiatric Inpatients

**DOI:** 10.3389/fpsyg.2019.00289

**Published:** 2019-02-21

**Authors:** Ali M. Molaie, Chih-Yun Chiu, Zara Habib, Igor Galynker, Jessica Briggs, Paul J. Rosenfield, Raffaella Calati, Zimri S. Yaseen

**Affiliations:** ^1^Department of Psychiatry, Mount Sinai Beth Israel, New York, NY, United States; ^2^Department of Psychiatry, Mount Sinai St. Luke's and Mount Sinai West, New York, NY, United States

**Keywords:** attachment, emotional pain, self-injurious behaviors, suicide, suicide attempts, inpatient

## Abstract

**Background:** Non-suicidal self-injury (NSSI) is a risk factor for suicide attempts (SA). Both attachment disturbances and cognitive and emotional problems (e.g., emotional pain) have been associated with SA history. This study sought to determine differential contributions of attachment styles and cognitive and emotional states associated with SA to lifetime NSSI occurrence among adults hospitalized for suicide risk.

**Sampling and Methods:** Adult psychiatric inpatients (*n* = 200) were assessed for attachment style, cognitive and emotional states, and lifetime NSSI within 72 h of hospitalization. Binary logistic regression and mediation analyses were performed.

**Results:** Preoccupied attachment and emotional pain at admission were independently associated with lifetime NSSI. Emotional pain partially mediated the relationship between preoccupied attachment and lifetime NSSI.

**Limitations:** The cross-sectional nature of the study and the use of a dichotomous (yes/no) measure of NSSI, not specifically designed for its assessment.

**Conclusions:** Preoccupied attachment and emotional pain are associated with NSSI and may be useful targets for assessing risk of NSSI.

## Introduction

Non-suicidal self-injury (NSSI) is defined as deliberate self-inflicted harm to one's body tissue without the intent to die and for purposes that are not socially sanctioned (Turecki and Brent, 2016)—a prevalent and debilitating public health concern across demographic groups and clinical populations (Heath et al., 2009; Klonsky, 2011). Burning, cutting, scratching, and self-hitting are common behaviors in NSSI (Cipriano et al., 2017). NSSI is currently a diagnostic criterion for borderline personality disorder, although recent conceptualizations suggest that NSSI occurs across multiple clinical disorders and may occur independent of any psychiatric comorbidities (Zetterqvist, 2015). Given the prevalence and trans-diagnostic nature of NSSI, a workgroup published a proposal in the *Diagnostic and Statistical Manual of Mental Disorders* 5 (DSM-5) to include *NSSI disorder* under conditions for further study (American Psychiatric Association, 2013).

Although the majority of NSSI research has been conducted on adolescent populations, up to 21% of adult psychiatric inpatients engage in NSSI (Briere and Gil, 1998). NSSI is particularly prevalent among psychiatric inpatients with a history of suicide attempts (SA) (Langbehn and Pfohl, 1993; Andover and Gibb, 2010). The relationship between NSSI and SA is complex. Although frequently considered distinct entities on the basis of the presence/absence of suicidal intention, studies consistently demonstrate high levels of comorbidity between non-suicidal and suicidal self-injury (Zetterqvist, 2015). Furthermore, research shows that common heritable factors are shared by NSSI and suicidal ideation/SA (Maciejewski et al., 2014). Indeed, a substantial proportion of individuals who attempt suicide report NSSI (Franklin and Nock, 2016), and a history of NSSI increases risk of suicide following or during psychiatric inpatient treatment (King et al., 2001; Hunt et al., 2007). This association may be expected as NSSI is commonly understood as a maladaptive affect regulation strategy (Klonsky, 2009; Nock, 2009) that may increase risk of suicide through an acquired capability for lethal self-harm (Joiner et al., 2012; Chu et al., 2018).

Evidence suggests that additional risk factors, both environmental and individual, may play a role in the etiology of NSSI. Disturbances in attachment have been linked to both NSSI (Adam, 1994; Critchfield et al., 2008) and suicidal behaviors (Adam et al., 1996; Lessard and Moretti, 1998; Stepp et al., 2008). Attachment theory suggests that experiences with caretakers beginning in infancy are internalized as working models of self and other (Griffin and Bartholomew, 1994) that profoundly influence an individual's ability to regulate affect (Schore, 2015) and manage relationships (Levy and Blatt, 1999; Crowell et al., 2008). Four primary adult attachment styles have been described in the literature: secure, preoccupied (also referred to as anxious), dismissing, and fearful, the latter three of which are considered variants of insecure attachment (Bartholomew and Horowitz, 1991). Characteristics associated with preoccupied attachment appear to be particularly related to NSSI. Increased levels of self-blame and self-criticism have been noted in individuals with preoccupied attachment (Bartholomew and Horowitz, 1991). Recent studies support the role of negative associations with the self in motivating NSSI through the belief that one deserves punishment (Franklin and Nock, 2016). Moreover, preoccupied or anxiously attached individuals tend to bolster their unstable sense of self-worth through seeking excessive reassurance of intimacy in personal relationships, leaving them vulnerable to extreme distress when their intimacy needs are not met (Griffin and Bartholomew, 1994). Thus, individuals with preoccupied attachment disturbances may be more likely to engage in NSSI in the context of acute affective dysregulation, specifically in response to interpersonal stress. Accordingly, several studies have found a direct effect of insecure or anxious (i.e., preoccupied) attachment style on NSSI (van der Kolk et al., 1991; Gratz et al., 2002; Stepp et al., 2008; Martin et al., 2011).

Much of affect regulation is learned through the formative attachment experiences in infant-caregiver dyads (Mikulincer et al., 2003; Mikulincer and Shaver, 2008). Acute cognitive and emotional states associated with imminent suicide risk may play a role in the development and maintenance of NSSI (Cuenca, 2013). In particular, relief of emotional pain has been shown to serve a powerful reinforcing function for NSSI (Brown et al., 2002; Nock and Prinstein, 2004). Likewise, recent work has highlighted the importance of emotional pain and entrapment in the etiology of SA (Shneidman, 1993, 1999; Orbach et al., 2003; Rasmussen et al., 2009; Yaseen et al., 2012, 2016; Ducasse et al., 2017; Galynker et al., 2017). Thus, the same acute emotional states associated with SA may also be related to NSSI.

Current research suggests that regulation of emotional states may mediate the relation between attachment insecurity and NSSI in young adults (Kimball and Diddams, 2007). Some researchers posit that individuals with insecure attachment styles may be less able to regulate painful affective states (Schaffer, 1993) and are more prone to experiencing intolerable emotional pain, leading to affect regulation in the form of NSSI (Cuenca, 2013). Preoccupied attachment may therefore increase susceptibility to experiences of emotional pain, which would thus mediate its relationship with NSSI.

The high rate of NSSI among adult psychiatric patients, coupled with the strong association between NSSI and suicide, makes this population particularly vulnerable (Pompili et al., 2015). While several studies have examined the relationship between insecure attachment and affective state factors on the presence of NSSI, to our knowledge no investigations have analyzed the interrelation of the two factors in an adult suicide risk sample. Thus, we sought to address this gap in the literature by examining the association between attachment characteristics, acute cognitive and emotional states previously associated with short-term suicide risk, and NSSI in a sample of adult patients hospitalized for acute suicide risk.

Based on the existing literature, we first hypothesized that preoccupied attachment would be associated with lifetime NSSI among attachment styles (secure, preoccupied, dismissing, fearful). Second, we hypothesized that emotional pain would be associated with lifetime NSSI among the acute cognitive and emotional states previously associated with acute suicide risk (entrapment, panic/dissociation, ruminative flooding, fear of dying, emotional pain). Finally, we hypothesized that emotional pain would mediate the relationship between preoccupied attachment and NSSI. We tested these hypotheses cross-sectionally, using presence of lifetime NSSI as the outcome.

## Methods

### Participants and Setting

The study was approved by the Institutional Review Board of a downtown Manhattan full-service community and tertiary care teaching hospital. The hospital houses 92 psychiatric inpatient unit beds, and serves a diverse urban population. All participants were interviewed within 72 h of admission in one of three psychiatric inpatient units in the hospital, and were recruited from April 2013 through July 2015 as part of a larger study examining correlates of imminent suicide risk in psychiatric inpatients. Research staff asked the treating clinical staff for referrals to appropriate patients.

### Inclusion and Exclusion Criteria

Males and females 18–65 years of age admitted to an inpatient psychiatric unit for clinically determined suicide risk based on presentation with suicidal ideation or SA preceding hospital admission were included. Patients exhibiting intellectual disability, cognitive impairment, severe psychotic symptoms, such as disorganization, or linguistic limitations precluding understanding of the consent or research questions, or significant medical or neurological disease or possible delirium were excluded from the study. Additionally, patients without current residence or contact information, and at least one collateral contact to allow for follow-up interviews were excluded from participation. A total of 200 patients qualified for study inclusion, provided informed consent, and provided sufficient information to the researchers for use in the study. Participants were reimbursed $50 for their participation.

### Clinical Assessment

Participants were approached and completed the study battery within ~3 days of admission to the inpatient unit. The assessment was performed by trained research assistants under the supervision of experienced psychiatrists with a research background.

Clinical diagnoses were made by the board certified attending psychiatrists of the inpatient units within an Accreditation Council for Graduate Medical Education (ACGME) accredited teaching hospital. Diagnoses were extracted from the electronic medical records of the participating patients and grouped into four mutually exclusive categories following our previously used methodology (Yaseen et al., 2016). DSM-IV Axis I diagnoses were coded as follows: (1) Psychotic spectrum disorders (comprising diagnoses of schizophrenia, schizoaffective disorder, and psychotic disorder not otherwise specified); (2) Bipolar spectrum disorders (comprising diagnoses of bipolar disorders, type I, II, or not otherwise specified); (3) Unipolar depressive disorders (comprising diagnoses of major depressive disorder, major depressive episode, and depression not otherwise specified); or (4) All other disorders (mainly comprising diagnoses of adjustment disorder and mood disorder not otherwise specified). In addition, the presence or absence of a substance use disorder, which could be comorbid with the primary psychiatric diagnosis, was extracted from the electronic medical record. This diagnostic categorization was used since these were the most common diagnoses among our inpatient population and they require different treatments. We have used this classification scheme in several of our prior papers (Li et al., 2017, 2018).

### Measures

The study team interviewed participants and administered a psychological test battery including measures of lifetime NSSI, attachment style, and cognitive and emotional states associated with acute suicide risk.

Adult attachment styles were assessed at admission using the Relationship Scales Questionnaire (RSQ) (Bartholomew and Horowitz, 1991), a 30-item self-report measure with responses rated on a 5-point Likert scale. Four attachment styles are assessed: (1) Secure: a pattern of comfort with intimacy and autonomy combined with positive evaluations of self and others; (2) Fearful: fear of emotional closeness combined with negative evaluations of self and others; (3) Dismissing: restricted emotionality in relationships combined with positive evaluations of self and negative evaluations of others; and (4) Preoccupied: a pattern of over-dependence on an attachment figure and fear of abandonment, combined with negative evaluations of self and positive evaluations of others (Griffin and Bartholomew, 1994; Ciechanowski et al., 2003).

Acute cognitive and emotional states associated with suicide risk were assessed at admission using the 49-item self-report Suicide Crisis Inventory (SCI) (Galynker et al., 2017). The SCI comprises five subscales: (1) Entrapment: feelings of desperation, hopelessness, and an urge to escape the situation; (2) Panic/Dissociation: altered sensorium and derealization associated with panic and anxiety; (3) Ruminative Flooding: uncontrolled, racing, and rigid negative thoughts as well as headache and head pressure; (4) Emotional Pain: feelings of intolerable agonizing mental pain and anguish, associated but distinct from symptoms of depression; and (5) Fear of Death: fear of dying and worry of sudden death, associated with but distinct from panic and anxiety.

NSSI was assessed using a dichotomous (yes/no) item of the Columbia Suicide Severity Rating Scale (C-SSRS) (Posner et al., 2011).

### Data Analysis

Each analysis was preceded by a test of its underlying assumptions. Where normality was violated, non-parametric analogs for parametric tests were used. Review of the Shapiro-Wilk test for normality showed that no subscale of both the RSQ and SCI was normally distributed. Thus, the Mann-Whitney *U* Test and Spearman's Correlations were used for bivariate analyses involving these measures. Binary logistic regression was used for multivariate analyses. Variance inflation factor (VIF) values (multiple linear regression analyses) were calculated to assess multicollinearity.

To test the hypotheses that emotional pain and preoccupied attachment style would be associated with NSSI, scores on the RSQ and SCI were compared between participants who did and did not endorse lifetime NSSI. The independent contributions of each subscale of the RSQ and SCI to lifetime NSSI were then examined using binary logistic regression. Furthermore, to test the hypothesis that emotional pain at admission would mediate the relationship between preoccupied attachment style and lifetime NSSI, mediation analysis was conducted.

The significance criterion for mediation effects was set at α ≤ 0.05, assessed with bootstrapped 95% confidence intervals (CIs) for indirect effects. The Sobel Z score was used as a measure of effect size. All statistical analyses were conducted using SPSS version 25, and mediation analyses were conducted using the PROCESS macro for SPSS, version 2.13 (43).

## Results

### Socio-Demographic and Clinical Characteristics

The final sample consisted of 200 participants. The demographics are presented in Table 1. Briefly, approximately half (53.5%) of the patients were females, with a mean age of 35.4 ± 13.4. Slightly over half of participants were diagnosed with a mood disorder (55%), while the rest were diagnosed with psychotic (10%) or other (35%) disorders.

**Table 1:** Sample characteristics (NSSI, non-suicidal self-injury; SA, suicide attempt; SB, suicidal behavior; SUD, substance use disorder).

	**Whole sample (*n* = 200)**	**Lifetime NSSI (*n* = 97)**	**No lifetime NSSI (*n* = 103)**	**Chi^**2**^/*t*-test**	**d.f**.	***p***
**SOCIO-DEMOGRAPHICS—*****N*** **(%) OR MEAN [SD], AS APPROPRIATE**						
Gender (female)	107 (53.5)	53 (54.6)	54 (52.4)	2.37	2	0.31
Ethnicity (Non-Hispanic)	132 (66.0)	61 (62.9)	71 (68.9)	0.81	1	0.37
Age	35.4 [13.4]	31.4 [12.2]	39.1 [13.5]	−4.2	198	< 0.0001
Annual income (< $20K)	101 (50.5)	43 (46.2)	58 (56.3)	5.26	5	0.38
Years of education	13.7 [2.9]	13.7 [3.1]	13.8 [2.8]	−0.19	198	0.85
**SELF-INJURIOUS BEHAVIORS—*****N*** **(%)**						
Lifetime SA	139 (69.5)	74 (76.3)	65 (63.1)	4.09	1	0.04
SA leading to admission	58 (29.0)	33 (34.0)	25 (24.3)	2.31	1	0.13
SB leading to admission	70 (35.0)	39 (40.2)	31 (30.1)	2.24	1	0.13
**CLINICAL FEATURES—*****N*** **(%)**						
**Primary diagnosis**						
Psychotic spectrum disorders	20 (10.0)	9 (9.3)	11 (10.7)	0.42	3	0.94
Bipolar spectrum disorders	26 (13.0)	14 (14.4)	12 (11.7)			
Unipolar depressive disorders	84 (42.0)	40 (41.2)	44 (42.7)			
Other^a^	70 (35.0)	34 (35.1)	36 (35.0)			
Psychotic symptoms	33 (16.5)	17 (17.5)	16 (15.5)	0.14	1	0.70
Ethanol use disorder	75 (37.5)	35 (36.1)	40 (38.8)	0.16	1	0.69
Illicit SUD	97 (48.5)	54 (55.7)	43 (41.7)	3.88	1	0.049
Any SUD	120 (60.0)	64 (66.0)	56 (54.4)	2.81	1	0.09

a *Mood disorder not otherwise specified, adjustment disorder, substance-induced disorders, personality disorder only, anxiety disorders*.

Roughly half of our sample (48.5%) endorsed lifetime NSSI. Patients with a NSSI lifetime history did not differ on the majority of the socio-demographic and clinical variables, with the exception of age (they were younger), lifetime SA, and illicit substance use disorder (both associated with higher rates of NSSI).

### Attachment Styles and SCI Components

For scores at RSQ and SCI see Table 2. Patients with lifetime NSSI scored higher than patients without it on three RSQ subscales: (1) preoccupied (*p* < 0.0001), (2) dismissing (*p* = 0.01), and (3) fearful (*p* = 0.001). Patients with lifetime NSSI scored lower than patients without it on the secure attachment subscale (*p* = 0.01). To control for inter-correlations among subscales, we conducted a binary logistic regression using all four attachment styles as predictors of lifetime NSSI. Preoccupied attachment style was the sole significant independent predictor (B = 0.14, AOR = 1.15, *p* = 0.006) (Table 3). The variance inflation factor (VIF) values of the four attachment styles were all < 1.5, indicating low multicollinearity.

**Table 2:** Sample scores at the Relationship Scales Questionnaire and Suicide Crisis Inventory.

	**Whole sample (*n* = 200)**	**Lifetime NSSI (*n* = 97)**	**No lifetime NSSI (*n* = 103)**	***p***^**a**^
**RELATIONSHIP SCALES QUESTIONNAIRE—MEAN [SD]**				
Secure	14.1 [3.6]	13.4 [3.3]	14.7 [3.8]	0.01
Preoccupied	12.4 [3.2]	13.3 [3.4]	11.6 [2.7]	< 0.0001
Dismissing	17.6 [3.5]	18.1 [3.6]	17.0 [3.3]	0.01
Fearful	13.0 [4.0]	13.9 [4.2]	12.1 [3.6]	0.001
**SUICIDE CRISIS INVENTORY—MEAN [SD]**				
Total Score	103.3 [46.2]	113.4 [40.0]	93.8 [49.7]	0.004
Entrapment	32.2 [14.5]	35.3 [12.8]	29.4 [15.4]	0.009
Panic/Dissociation	11.3 [9.1]	12.1 [8.7]	10.5 [9.4]	0.09
Ruminative flooding	15.9 [8.1]	17.8 [7.4]	14.2 [8.4]	0.002
Fear of dying	5.0 [3.8]	5.1 [3.7]	4.9 [4.0]	0.57
Emotional pain	9.5 [5.2]	10.9 [4.8]	8.3 [5.2]	0.001

a *Mann-Whitney U Test*.

**Table 3:** Binary logistic regression: Admission Relationship Scales Questionnaire subscales prediction of lifetime Non-Suicidal Self-Injury (AOR, adjusted odds ratio).

**Variable**	**B**	**S.E**.	***p***	**AOR**
Secure	−0.046	0.048	0.339	0.955
Preoccupied	0.139	0.050	0.006^**^	1.15
Dismissing	0.019	0.051	0.706	1.02
Fearful	0.066	0.047	0.155	1.07

** *p < 0.01*.

Patients with lifetime NSSI scored higher than patients without it on SCI total score (*p* = 0.004), and on three SCI subscales: (1) entrapment (*p* = 0.009), (2) ruminative flooding (*p* = 0.002), and (3) emotional pain (*p* = 0.001). There were no differences in scores on panic/dissociation and fear of dying subscales between the two groups. To control for inter-correlations among subscales, we conducted a binary logistic regression analysis using all five SCI variables as predictors of lifetime NSSI. Emotional pain was the sole significant independent predictor (B = 0.08, AOR = 1.09, *p* = 0.048) (Table 4). The VIF values of the five SCI subscales were < 2.5, indicating low multicollinearity.

**Table 4:** Binary logistic regression: Admission Suicide Crisis Inventory subscales prediction of lifetime Non-Suicidal Self-Injury (AOR: adjusted odds ratio).

**Variable**	**B**	**S.E**.	***p***	**AOR**
Entrapment	0.007	0.017	0.671	01.01
Panic/Dissociation	−0.015	0.023	0.515	0.985
Ruminative Flooding	0.047	0.028	0.098	1.05
Fear of Dying	−0.080	0.051	0.115	0.923
Emotional Pain	0.085	0.043	0.048^*^	1.09

* *p < 0.05*.

Among patients who endorsed lifetime NSSI, preoccupied attachment style was correlated with emotional pain at admission (rs = 0.21, *p* = 0.003).

### Relations Between Preoccupied Attachment, Emotional Pain at Admission, and Lifetime NSSI

In mediation analysis, acute emotional pain was a significant partial mediator of the relationship between preoccupied attachment style and lifetime NSSI (B = 0.031, Bootstrapped 95% CI = 0.008–0.070) (Table 5).

**Table 5:** Mediation analysis on lifetime Non-Suicidal Self-Injury.

**Predictor**	**Mediator**	**Total effect**	***p***	**Direct effect**	***p***	**Indirect effect**	**Bootstrapped 95% CI**	**Sobel Z**
Preoccupied attachment	Emotional Pain at admission	0.174	< 0.001^***^	0.152	0.002^**^	0.031	0.008–0.070	2.067^*^

* *p < 0.05*.

** *p < 0.01*.

*** *p < 0.001*.

## Discussion

In this study of adult psychiatric inpatients hospitalized for suicide risk, half of the sample (48.5%) endorsed lifetime NSSI, highlighting the prevalence of this behavior among a high risk population. Moreover, contrary to popular conceptions, NSSI was equally prevalent among men and women in this sample. Few studies have examined risk factors for NSSI in adult samples (Kapur et al., 2013). To our knowledge, this is the first paper to analyze the relationship between attachment styles, acute cognitive and emotional states, and NSSI among high suicide risk adult psychiatric inpatients.

In support of our first hypothesis, we found that preoccupied attachment style was the sole independent predictor of lifetime NSSI among attachment disturbances. This finding parallels prior research suggesting a link between preoccupied attachment and NSSI, particularly in adolescent populations (Wright et al., 2005; Stepp et al., 2008), but also in adults (Critchfield et al., 2008). Individuals with preoccupied attachment, characterized by a sense of unworthiness or unlovability of the self, and a positive working model of the other, may favor coping behaviors, such as NSSI that are hurtful to self but elicit rescue from caregivers (Griffin and Bartholomew, 1994; Bolen et al., 2013) or that instantiate negative beliefs about oneself (Franklin and Nock, 2016).

Of the acute cognitive and emotional states examined, emotional pain at admission was the sole independent predictor of lifetime NSSI. Hence, the key role of emotional pain is not only traceable in suicidal ideation and SA (Ducasse et al., 2017) but in NSSI as well. The observed data highlight the importance of identifying the automatic negative reinforcement function of NSSI that serves to relieve negative affective states, such as emotional pain (Nock and Prinstein, 2004). Although entrapment and ruminative flooding were each individually predictive of NSSI in univariate analyses, they did not remain significant in the binary logistic regression. Hence, in our sample intolerable emotional pain appeared to be more closely associated with lifetime NSSI in comparison to other acute cognitive and affective states. Although the SCI subscales are intercorrelated, the low VIF values suggest that this did not unduly affect the reliability of the multivariable logistic regression model.

We also found preoccupied attachment style to be correlated with emotional pain. This result is in agreement with literature documenting the interplay between preoccupied attachment and negative emotional reactivity (Levy et al., 2005).

In support of our third hypothesis, emotional pain at admission partially mediated the relationship between preoccupied attachment style and lifetime NSSI. NSSI may serve as a potent coping behavior for regulation of negative affective states, such as unbearable emotional pain, for individuals with relationship histories characterized by instability (Linehan, 1993; Chapman et al., 2006; Kleindienst et al., 2008).

From a neurobiological perspective, the endogenous opioid system plays a role in pain threshold and perception and is a good candidate for involvement in NSSI, a behavior often associated with the need to feel pain and/or to relieve emotional tension. Patients with a history of NSSI were found to have significantly lower levels of cerebrospinal fluid (CSF) beta-endorphin and met-enkephalin when compared with non-NSSI patients (Stanley et al., 2010). Moreover, the opioid system seems to be implicated in social pain as well (Lutz et al., 2018). A history of chronic stress may lead to a dulled endogenous opioid response to acute stress, possibly increasing vulnerability to emotional pain. NSSI may be an effort at increasing the endogenous opioids to re-establish homeostasis (Stanley et al., 2010). Future studies should explore possible treatment approaches that can modulate opioid system function in patients with NSSI (Turner et al., 2014).

Although beyond the scope of this report, many other factors contribute to suicide and NSSI risk, both additively and in interaction with the factors examined here, and would deserve attention. For example, sleep disturbances, and in particular insomnia (Pompili et al., 2013), may both mark distress and further reduce distress tolerance and impulse inhibition, leading to increased risk.

### Clinical Implications

Taken together, our results suggest that primary and long-term secondary prevention strategies for NSSI may be improved by focusing on attachment and interpersonal dysfunction (Linehan, 1993; Yeomans et al., 2013). In particular, patients with a preoccupied attachment style trust in others, but not in themselves and can benefit from interventions aimed at improving their attachment style. Furthermore, this study examined cognitive and emotional states including entrapment and emotional pain that have been reported to predict acute risk of SA in psychiatric populations (Hendin et al., 2010; Yaseen et al., 2014). Similar affective precursors may underlie NSSI, in particular emotional pain, and preoccupied attachment may increase susceptibility to them. Hence a thorough attention to both acute affective states and broader relational factors could make the difference in the clinical approach to patients with a lifetime history of NSSI.

### Limitations

The cross-sectional nature of the study limits the possibility to infer causal relationships. A dichotomous (yes/no) measure was used to assess NSSI, thus precluding more detailed investigation of frequency and severity of NSSI; moreover, the C-SSRS was not specifically designed to assess NSSI. Moreover, desirability biases may have influenced patient reports in the hospital setting. Finally, voluntary participation in the study may have introduced selection bias.

### Conclusion

This study provides evidence that preoccupied attachment style and emotional pain are associated with NSSI in adult psychiatric patients acutely hospitalized for suicide risk, with emotional pain partially mediating the effect of preoccupied attachment on NSSI.

## Author Contributions

AM and C-YC performed the first literature search, the first statistical analyses, and wrote a first draft of the manuscript. ZH revised the paper and performed an updated literature search. JB contributed to the writing of the paper. RC performed further statistical analyses and contributed to the writing of the last version of the manuscript. ZY was responsible of data collection, proposed the topic, closely supervised AM and C-YC in the manuscript preparation and revised the manuscript. IG and PR were responsible of data collection and supervised the manuscript writing.

### Conflict of Interest Statement

The authors declare that the research was conducted in the absence of any commercial or financial relationships that could be construed as a potential conflict of interest.
